# Osteophytenbedingtes Impingement verringert die Beweglichkeit bei in „Humpback“-Deformität fehlverheilter Skaphoidrekonstruktion

**DOI:** 10.1007/s00113-020-00825-3

**Published:** 2020-05-30

**Authors:** P. Moog, M. K. Cerny, D. Schmauss, J. Betzl, S. Löw, H. Erne

**Affiliations:** 1 Klinik und Poliklinik für Plastische Chirurgie und Handchirurgie, Klinikum rechts der Isar, Technische Universität München, Ismaninger Str. 22, 81675 München, Deutschland; 2 Abteilung für Plastische, Rekonstruktive und Ästhetische Chirurgie, Ospedale Regionale di Lugano, Lugano, Schweiz; 3 Praxis für Handchirurgie und Traumatologie, Bad Mergentheim, Deutschland

**Keywords:** Humpback-Deformität, Pseudarthrose, Skaphoidfraktur, Skaphoidrekonstruktion, Humpback deformity, Pseudarthrosis, Scaphoid fracture, Scaphoid reconstruction

## Abstract

**Hintergrund:**

Der Goldstandard in der Therapie der skaphoidalen Pseudarthrose ist die Reduktion und Versorgung mit Beckenkamminterponat und Herbert-Schraube, um die häufig beobachtete Humpback-Deformität zu reduzieren. Diese Studie korreliert das Ausmaß der Humpback-Deformität nach Skaphoidrekonstruktion mit postoperativen klinischen und radiologischen Parametern.

**Material und Methoden:**

Zwischen 2008 und 2010 wurden 56 Patienten mit skaphoidaler Pseudarthrose operiert. Davon konnten 34 in diese retrospektive Studie eingeschlossen werden. Das durchschnittliche Nachuntersuchungsintervall betrug 7,3 Monate. Die Humpback-Deformität wurde entlang der Skaphoidlängsachse mittels Computertomographie (CT) beurteilt, während für das klinische Ergebnis der Disability of the Arm, Shoulder and Hand (DASH) Score sowie die Handkraft (Jamar), der Bewegungsumfang (RoM), Mayo Wrist Score (MWS) und andere Parameter verwendet wurden.

Die Patienten wurden in 2 Gruppen eingeteilt: 1. keine oder nur geringe Humpback-Deformität (<25°), 2. schwere Humpback-Deformität (>45°).

**Ergebnisse:**

Der Bewegungsumfang und die DASH Scores waren für die erste Gruppe etwas besser, während die zweite eine signifikant erhöhte Inzidenz für Osteophytenbildung (*p* < 0,05) und verringerten Bewegungsumfang (−16°) aufwies.

**Diskussion:**

Wir postulieren, dass der größte Nachteil einer nichtreduzierten Humpback-Deformität das häufigere Auftreten von Osteophyten im dorsalen Aspekt des Skaphoids ist. Dies kann in der Extension eine Einklemmung hervorrufen und somit signifikanten Einfluss auf den Bewegungsumfang des Handgelenks nehmen.

**Grad der Evidenz:**

III

## Abstract (en)

**Background:**

The gold standard in the treatment of scaphoid pseudarthrosis is reduction, interposition of an iliac crest graft and stabilization with a headless bone (Herbert) screw, aiming to reduce the frequently observed humpback deformity. This study correlated the extent of humpback deformity after scaphoid reconstruction to clinical and radiological postoperative parameters.

**Material and methods:**

Between 2008 and 2010 a total of 56 patients with scaphoid pseudarthrosis were surgically treated. Of the patients 34 could be included in this retrospective study. The average follow-up period was 7.3 months. The humpback deformity was evaluated by computed tomography (CT) scan performed along the long axis of the scaphoid. The disability of the arm, shoulder and hand (DASH) score, grip strength (Jamar), range of motion (RoM), Mayo wrist score (MWS) and other parameters were used to determine the clinical outcome.

The patients were divided into two groups: 1) no or only slight humpback deformity (<25°), 2) severe humpback deformity (>45°).

**Results:**

The RoM and DASH scores were slightly better for the first group. The second group had a significantly increased incidence of osteophyte formation (*p* < 0.05) and decreased RoM (−16°).

**Conclusion:**

It is postulated that the main disadvantage of an nonreduced humpback deformity is the increased occurrence of osteophyte formation in the dorsal aspect of the scaphoid. This can cause an impingement during extension and leads to a significant restriction of movement of the wrist.

## Hintergrund

Das Handgelenk, bestehend aus einer Vielzahl von ineinandergreifenden Knochen mit komplizierten Gelenkverbindungen, ist aus anatomischer und biomechanischer Sicht vermutlich das komplexeste Gelenk des menschlichen Körpers [1]. Die häufigsten Frakturen der Handwurzel sind die des Skaphoids, die 80–90 % aller Handwurzelknochenfrakturen ausmachen [2–4]. Diese treten mit einer Häufigkeit von 12,4/100.000 Frakturen und Jahr auf, meistens bei jungen Männern [4, 5]. Die Rate an Pseudarthrosen ist mit bis zu 10 % relativ hoch, selbst wenn die Fraktur adäquat versorgt wird [4]. Besonders dislozierte Frakturen scheinen zu Pseudarthrosen zu neigen, bedingt durch interfragmentäre Instabilität, retrograde vaskuläre Versorgung und Mangel an bindegewebiger Befestigungen auf einer hauptsächlich knorpeligen Oberfläche [6]. Skaphoidale Pseudarthrose führt zu abnormer Handgelenkkinematik und kann zu Kollaps der Handwurzelknochen und in der Folge degenerativer Veränderung des Handgelenks führen [4, 7]. Die meisten skaphoidalen Pseudarthrosen sind symptomatisch und weisen Humpback-Deformitäten (Dislokationsform mit Buckelbildung des Skaphoids) und dorsale interkalierte Segementinstabilitäten (DISI) auf [4]. Wenn sich die Fraktur im mittleren Drittel des Skaphoids befindet, ist die Pseudarthrose besonders häufig mit einer Humpback-Deformität assoziiert, die aus einem Flexionsmoment an der Frakturstelle mit begleitender Flexionsfehlstellung des distalen Fragments entsteht [8, 9]. Die konventionelle Therapie dieser Deformität besteht meist aus einem bikortikalen Beckenkamm-Wedge-Interponat mit interner Fixation mittels Herbert-Schraube [10]. Die Verwendung einer Herbert-Schraube hat dabei eine verbesserte karpale Stabilität gezeigt und in 71–100 % der Fälle die Knochenverbindung wiederhergestellt [10–14]. Wichtige Prinzipien, die die Therapie der skaphoidalen Pseudarthrose leiteten, waren Exzision, Korrektur der Gelenkfehlstellung (Wiederherstellung der Handwurzelknochenhöhe und -gelenkführung [12]), ein überbrückendes Knocheninterponat, mechanische Kompression, stabile Fixation [10–15] und Korrektur der häufigen Humpback-Deformität [12].

Diese Studie soll die Humpback-Deformität mit klinischen und radiologischen Parametern korrelieren und zeigte eine interessante Entdeckung, die die eingeschränkte Beweglichkeit erklären kann.

## Material und Methoden

### Demografie

Diese retrospektive Studie wurde im Einklang mit den bekannten ethischen Grundsätzen durchgeführt. Zwischen 2016 und 2018 wurden 56 Patienten mit skaphoidaler Pseudarthrose in unserem Zentrum operiert. 7 Patienten mit nichtverheiltem Skaphoid oder anderen eindeutigen Ursachen für einen eingeschränkten Bewegungsumfang (z. B. herausragende Schrauben) wurden aus dieser Studie ausgeschlossen. Ebenso wurden Humpback-Deformitäten, die im CT mit 25–45° vermessen wurden, exkludiert. Da in diesem Messbereich mögliche Fehler aufgrund von technischen Einzelheiten nicht eindeutig ausgeschlossen werden können, wollten wir so sicherstellen, dass nur zweifelslos normale (<25°) oder zweifelslos pathologische (>45°) Messwerte eingeschlossen werden.

**Figure 1:**
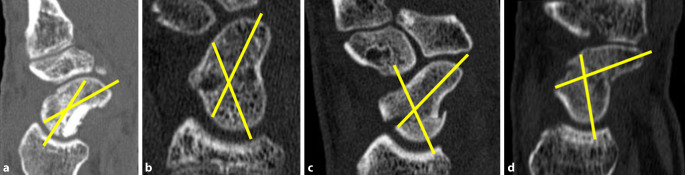

Es konnten insgesamt 34 Patienten in diese Studie eingeschlossen werden. Die Daten wurden retrospektiv aus den Aufzeichnungen der Nachuntersuchungen erhoben. Das durchschnittliche Patientenalter betrug 26 Jahre (Intervall: 16 bis 59 Jahre) mit 29 Männern und 5 Frauen. Das durchschnittliche Nachuntersuchungsintervall betrug 7,3 Monate. Die postoperative Humpback-Deformität wurde mittels CT-Bildgebung (Abb. 1) in der Achse des Skaphoids (lateraler interskaphoidaler Winkel) vermessen. Standardmäßige postoperative CT werden nach 8 Wochen sowie nach 6 Monaten angefertigt, um die Konsolidierung des zuvor frakturierten Skaphoids zu beurteilen.

Um Messfehler und die Intraobserver-Variabilität zu reduzieren, wurden die CT am gleichen Tag vom gleichen Untersucher begutachtet.

Die Patienten wurden anhand des gemessenen Winkels in 2 Gruppen eingeteilt: Gruppe I mit keiner oder nur einer geringen Humpback-Deformität bis zu 25° (10 Patienten) und Gruppe II mit schwerer Humpback-Deformität von mehr als 45° (24 Patienten).

Bei diesen Patienten wurden der DASH Score, der Mayo Wrist Score (MWS), die Handkraft (Jamar), der Bewegungsumfang (RoM) und, wenn vorhanden, die Ausbildung von Osteophyten ausgewertet.

### Operationstechnik

In allen Fällen wurde ein palmarer Zugangsweg gewählt. Die Sehne des M. flexor carpi radialis wurde nach radial gedrängt, um das Lig. radioscaphocapitatum freizulegen. Das Lig. radioscaphocapitatum wurde durchtrennt und später wieder genäht. Dann wurde die Pseudarthrose des Kahnbeins dargestellt. Es folgte das Débridement von sklerotischem Knochen und fibrösem Gewebe im Bereich der Pseudarthrose. Die Defektzone wurde mit Beckenkammspongiosa, in der „Press-fit“-Technik gefüllt sowie eine K‑Draht-geführte Osteosynthese des Kahnbeins mit einer kanülierten, kopflosen Doppelgewindeschraube nach Herbert durchgeführt. Es folgten der schichtweise Wundverschluss sowie eine Immobilisation mittels Unterarmhandgelenkschiene mit Daumengrundgelenkeinschluss für 8 Wochen.

### Disability of Arm, Shoulder and Hand Score

Der DASH Score, Version 2.0. (1997) [16] wurde verwendet, um das subjektive Funktionalitätsempfinden der Patienten zu quantifizieren.

### Mayo Wrist Score

Der MWS, wird standardmäßig zur Beurteilung von Einschränkungen und Funktionalität des Handgelenks sowie Schmerzen, Bewegungsumfang und Handkraftmessung eingesetzt [17].

### Messung der Handkraft

Die objektive Handkraftmessung wurde sowohl an der gesunden als auch an der operierten Hand mittels Jamar-Dynamometer durchgeführt, wobei die Kraft des Faustschlusses während dreier Wiederholungen gemessen wurde. Die Ergebnisse wurden jeweils zwischen der betroffenen und der gesunden Hand verglichen und als Prozentwert angegeben, die Dimension in Kilogramm.

### Messung des Bewegungsumfangs

In unseren Nachuntersuchungen wurde nur der passive Bewegungsumfang gemessen, der bei vom Untersucher geführten Bewegungen erreicht werden konnte. Dabei wurden jeweils 3 Werte analog zur Neutral-Null-Methode notiert und die Werte der maximalen Exkursionen addiert und als Summe (entsprechend dem maximalen Bewegungsumfang) notiert.

### Beurteilung der Osteophytenbildung

Die postoperativen CT wurden auf die mögliche Ausbildung von Osteophyten hin untersucht, wobei deren Schweregrad mitbeurteilt wurde. Um Messfehler zu reduzieren, wurden die Bilder von einem Untersucher am gleichen Tag begutachtet. Als Osteophyt wurde jeder in der CT-Bildgebung neu aufgetretene Osteophyt, der größer als 1 mm ist, gewertet.

### Statistik

Zur statistischen Analyse wurde der t‑Test für 2 Gruppen mittels SPSS 14 verwendet. Dabei wurden die Daten als Mittelwert ± Standardabweichung notiert. Die Wahrscheinlichkeit eines Fehlers 1. Art wurde bei 5 % (α = 0,05) festgelegt, außer wenn dies spezifisch anders angegeben wird.

## Ergebnisse

70 % der Patienten wiesen eine postoperative Humpback-Deformität von mehr als 45° (Gruppe II) nach einer durchschnittlichen Nachuntersuchungszeit von 7,3 Monaten auf.

**Figure 2:**
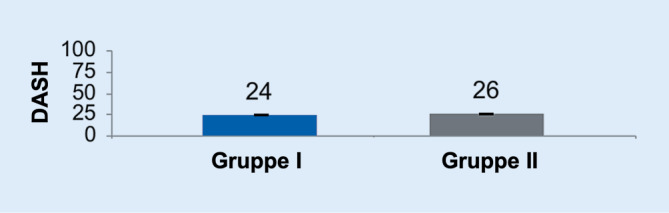

Der durchschnittliche DASH Score der Gruppe I betrug 24 Punkte, der der Gruppe II 26 Punkte (Abb. 2). Dies ist statistisch nicht signifikant (*p* > 0,05), ist jedoch gut vereinbar mit dem postoperativen klinischen Ergebnis beider Gruppen.

**Figure 3:**
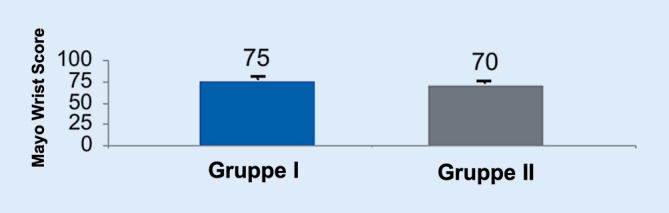

Zudem wurden das Outcome der Patienten mittels MWS (Abb. 3) beurteilt, wobei sich 75 Punkte der Gruppe I und 70 Punkte in Gruppe II (*p* > 0,05) zeigten. Dies zeigt ebenso, dass das postoperative Ergebnis für beide Gruppen vergleichbar gut war.

**Figure 4:**
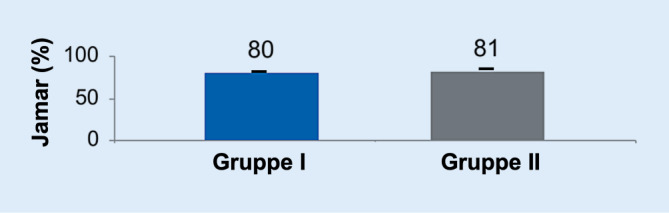

Die Handkraft (Abb. 4), die mit dem Jamar-Dynamometer gemessen, mit der gesunden Gegenseite verglichen und in Prozent angegeben wurde, zeigte für beide Gruppen fast identische Ergebnisse (*p* > 0,05).

**Figure 5:**
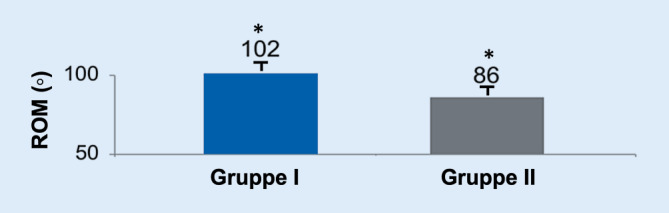

Der Bewegungsumfang zeigte größere Unterschiede zwischen beiden Gruppen: Während Gruppe I einen gesamtem Bewegungsumfang (Extension plus Flexion) von 102° zeigte, waren es nur 86° in Gruppe II (Abb. 5), was eine statistisch signifikante Differenz von 16° zwischen beiden Gruppen ergibt (*p* < 0,05). Zudem zeigte sich ein nichtsignifikanter Trend, dass die Beweglichkeit im zeitlichen Verlauf schlechter wird.

**Figure 6:**
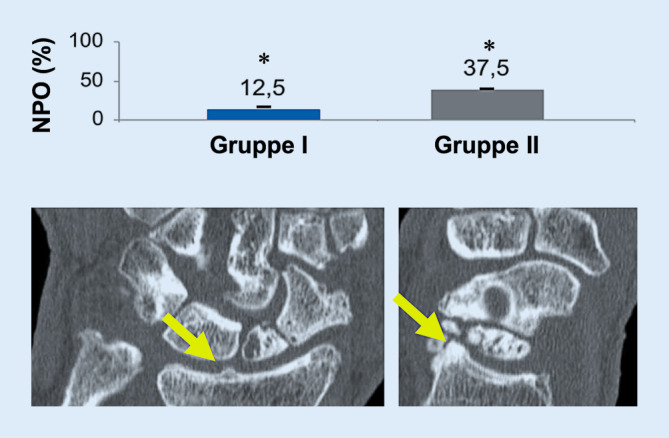

Um die Anzahl an Osteophyten zu quantifizieren, wurden CT-Bilder ausgewertet (Abb. 6). Ein Patient aus Gruppe I und 9 Patienten aus Gruppe II zeigten dabei Osteophyten von mehr als 1 mm Größe (*p* < 0,05).

## Diskussion

Die fehlende Konsolidierung einer Skaphoidfraktur kann zu Fehlstellungen der Handwurzelknochen und fortschreitender radiokarpaler Arthrose führen [7, 18–20]. Eine unzureichende skaphoidale Reposition kann eine Humpback-Deformität hervorrufen, was zu Verschiebungen im Frakturspalt und der Ausbildung der gezeigten Osteophyten führen kann. Ziele der Therapie sind daher die Reduktion der Deformität, Rekonsolidierung und Prävention einer Arthrose [10]. Die häufigste chirurgische Herangehensweise ist die Verwendung eines kompressionsresistenten Kortikalis-Spongiosa-Interponats, das typischerweise aus dem Beckenkamm gehoben und nach Transplantation intern fixiert wird [12].

In dieser Studie wurde der Einfluss des Ausprägungsgrades der Humpback-Deformität auf das klinische Ergebnis untersucht. 70 % der Patienten wiesen dabei eine Deformität von mehr als 45° (Gruppe II) nach einer Nachuntersuchungszeit von ca. 7,3 Monaten auf. Wir konnten zeigen, dass die Handkraft der betroffenen Hand in beiden Gruppen fast identisch (80 %) war. Dies ist gut mit anderen Studien vereinbar, die eine deutliche postoperative Verbesserung der Handkraft bis zu 79–104 % zeigten [12–14, 21, 22]. Die DASH Scores beider Gruppen waren ebenfalls vergleichbar (Gruppe I: 24 Punkte, Gruppe II: 26 Punkte). Cohen et al. (2013) führten eine offene Reduktion durch einen anterioren Zugang bei 12 Patienten durch, wobei sie die Deformität korrigierten und eine Distal-nach-proximal-Schraube platzierten [12]. Dann füllten sie den entstehenden Defekt mit einem autogenen Spongiosaspan aus dem ipsilateralen distalen Radius auf, was zu einem postoperativen DASH Score von 4 ± 3 (Min.: 0, Max.: 9) und MWS von 88 ± 6 (Min.: 80, Max.: 100) nach einem Mindestnachuntersuchungsintervall von 2 Jahren führte [12].

Diese Ergebnisse sind besser als unsere, wenn man die Scores vergleicht. Jedoch ist das Follow-up-Intervall – und damit der Heilungsprozess vermutlich auch – mehr als doppelt so lang wie unseres.

In unserer Studie schien Gruppe II (>45°) einen signifikant reduzierten Bewegungsumfang (*p* < 0,05) sowie eine signifikant erhöhte Inzidenz von Osteophyten in der CT-Bildgebung (*p* > 0,05) aufzuweisen. Wir vermuten, dass der eingeschränkte Bewegungsumfang durch das osteophytäre Impingement im dorsalen Aspekt des Skaphoids zu erklären ist. Zudem erwarten wir, dass im zeitlichen Verlauf die Zunahme der Osteophytenzahl zu einer weiteren Bewegungseinschränkung führen würde. Diese Beobachtung erzeugte jedoch im klinischen Outcome keine Unterschiede zwischen beiden Gruppen, insbesondere in Bezug auf die Handkraft, den DASH Score und den MWS.

Es wurde oft beschrieben, dass je länger eine Humpback-Deformität besteht, desto wahrscheinlicher eine DISI-Deformität entstehen wird [8]. Die sog. Humpback- oder flektierte skaphoidale Pseudarthrose entwickelt sich aus chronischem hohlhandseitigen Knochenverlust sowie einer intraskaphoidalen Kollapsdeformität mit resultierender dorsaler Verkippung des Lunatums (DISI) [10], was zu einer Prädisposition des Skaphoids für das Versagen zukünftiger Rekonsolidierung führt [23].

Zudem stört eine fortbestehende DISI-Deformität den normalen Bewegungsablauf des Handgelenks, indem sie die Höhe des Skaphoids reduziert und die normalen anatomischen Relationen zwischen den Handwurzelknochen verändert [1, 24, 25].

Dieser anormaler Knochenkontakt kann dann langfristig arthrotische Veränderungen hervorrufen [1, 26].

In unserer Studie wurden weniger DISI-Deformitäten festgestellt. Das könnte darauf zurückzuführen sein, dass DISI-Deformitäten nur eine sekundäre Indikation skaphoidaler Malokklusion darstellen. Wenn eine SL-Bandverletzung vorliegt, wird eine Rekonstruktion empfohlen, solange diese machbar erscheint. Die DISI soll zu kinematischen Störungen des Handgelenks führen – unsere Daten deuten darauf hin, dass die Humpback-Deformität dies auch tut. Die Auswirkungen dessen treten dann möglicherweise durch die Bildung von Osteophyten in Erscheinung, die in der CT-Bildgebung auffallen können. Unsere Ergebnisse zeigen ebenso, dass der reduzierte Bewegungsumfang mit der Inzidenz von Osteophyten korreliert, der sich schließlich in der Bewegungsreduktion äußert.

Die Schwächen dieser Studie liegen in der Schwierigkeit, die Osteophyten akkurat zu quantifizieren, da die wahre Grenze des Skaphoids und die Osteophytenbasis schwer zu erkennen waren. Wir haben allerdings mindestens 9 Patienten in Gruppe II eindeutig identifizieren können.

Außerdem hatten wir für unsere retrospektive Studie ein relativ kurzes Nachuntersuchungsintervall. Das liegt darin begründet, dass wir retrospektiv nur auf die CT-Bildgebung zurückgreifen konnten, welche standardisiert nach 6 bis 9 Monaten zur Konsolidierungskontrolle durchgeführt werden. Daher können wir keine Aussage bezüglich möglicher Arthrose durch Gelenkinkongruenz im späteren Verlauf treffen. Zudem erwies sich die genaue Bestimmung des Skaphoidwinkels als schwierig. Bain et al. (1988) haben die Variabilität dreier Techniken zur Messung der Humpback-Deformität in 37 Skaphoiden mittels longitudinaler CT-Bildgebung bewertet: den lateralen intraskaphoidalen Winkel, den dorsalen kortikalen Winkel und das Höhen-Längen-Verhältnis [27]. Sie kamen zu dem Schluss, dass die Intraobserver-Reliabilität des intraskaphoidalen Winkels weniger gut, die des dorsal-kortikalen Winkels mittel bis sehr gut und die des Höhen-Längen-Verhältnisses exzellent wäre. Daher scheint dieses die bestreproduzierbare Methode zur Beurteilung der Humpback-Deformität zu sein [27]. Infolgedessen könnte man argumentieren, dass unsere Messmethode (der laterale intraskaphoidale Winkel) eine weitere Schwäche unserer Studie darstellt.

Wir legen dar, dass der hauptsächliche Nachteil einer Humpback-Deformität das häufigere Auftreten von Osteophyten im dorsalen Aspekt des Skaphoids darstellt, was vermutlich die Hauptursache für einen reduzierten Bewegungsumfang darstellt. Daher sollte das Ziel nach Möglichkeit eine anatomische Reposition sein. Die vorliegende Arbeit bietet also erstmalig einen Erklärungsansatz für Bewegungseinschränkungen bei Humpback-Deformität nach Skaphoidrekonstruktion.

## Fazit für die Praxis

Es gibt keinen Unterschied im klinischen Outcome zwischen keinen Humpback- (Gruppe I) und ausgeprägten Humpback-Deformitäten (Gruppe II) in Bezug auf Handkraft, Disabilities of Arm, Shoulder and Hand (DASH) Score und Mayo Wrist Score (MWS). Gruppe II wies allerdings einen signifikant reduzierten Bewegungsumfang (−16°) und eine signifikant erhöhte Inzidenz bei der Entstehung von Osteophyten im CT auf.

Daher sollte das Therapieziel möglichst eine anatomische Reposition der Deformität sein, um diese beiden Effekte zu reduzieren.

## Abkürzungen

CT: Computertomographie („computer tomography“)
DASH: Disability of Arm, Shoulder and Hand Score
DISI: Dorsale interkalierte Segmentinstabilität („dorsal intercalated segment instability“)
NPO: Anzahl der Patienten mit Osteophyten („number of patients with osteophytes“)
RoM: Bewegungsumfang („range of motion“)
